# Prediction of distant metastatic recurrence by tumor-infiltrating lymphocytes in hormone receptor-positive breast cancer

**DOI:** 10.1186/s12905-021-01373-7

**Published:** 2021-05-29

**Authors:** Koji Takada, Shinichiro Kashiwagi, Yuka Asano, Wataru Goto, Rika Kouhashi, Akimichi Yabumoto, Sae Ishihara, Tamami Morisaki, Masatsune Shibutani, Hiroaki Tanaka, Kosei Hirakawa, Masaichi Ohira

**Affiliations:** 1 Department of Breast and Endocrine Surgery, Osaka City University Graduate School of Medicine, 1-4-3 Asahi-machi, Abeno-ku, Osaka, 545-8585 Japan; 2 Department of Gastrointestinal Surgery, Osaka City University Graduate School of Medicine, 1-4-3 Asahi-machi, Abeno-ku, Osaka, 545-8585 Japan

**Keywords:** Hormone receptor-positive breast cancer, Distant metastasis, Recurrence, Tumor-infiltrating lymphocytes, Tumor microenvironment

## Abstract

**Background:**

Breast cancer subtypes are known to have different metastatic recurrence sites. Distant metastases are often observed during the post-operative course in patients with human epidermal growth factor receptor 2 (HER2)-enriched breast cancer and triple-negative breast cancer, but are relatively rare in those with hormone receptor-positive and HER2-negative (HR+/HER2−) breast cancer. Tumor-infiltrating lymphocytes (TILs) serve as an index to monitor tumor immune microenvironment and may possibly predict the prognosis and therapeutic effect in breast cancer. This study aimed to investigate the correlation between TIL density and recurrence site in HR+/HER2− breast cancer.

**Methods:**

In stages I–II of HR+/HER2− breast cancer patients who underwent surgery as the first treatment and received adjuvant endocrine therapy (except adjuvant chemotherapy), forty-two patients relapsed after surgery. TILs were evaluated using needle biopsy specimens for the diagnosis of breast cancer. Morphological assessment was conducted using conventional hematoxylin and eosin staining.

**Results:**

Six patients had no TILs density. In them, local recurrence was significantly less (*p* = 0.022), while distant metastases were significantly more (*p* = 0.015) compared to those in patients with TIL density. Therefore, for the prediction of distant metastases in HR+/HER2− breast cancer without chemotherapy, TILs could be used as predictors in univariate analysis (*p* = 0.015, odds ratio [OR] = 0.127), although not as independent factors (*p* = 0.285, OR = 0.144).

**Conclusions:**

Our findings indicate that TILs may predict distant metastatic recurrence in stages I–II of HR+/HER2− breast cancer in patients who do not undergo chemotherapy.

**Supplementary Information:**

The online version contains supplementary material available at 10.1186/s12905-021-01373-7.

## Background

Cancer, even when detected early and successfully surgically resected, the risk of its recurrence persists [1–3]. Breast cancer often has local recurrence and axillary lymph node metastasis, although distant metastases, such as to the bone, lungs, and liver, may occur. Imaging procedures, such as computed tomography (CT), ultrasonography (US), and bone scintigraphy, are necessary to detect distant metastases sub-clinically. However, some prospective studies have shown early detection of distant metastases to not affect prognosis, and routine examination is not recommended in such cases [4–6]. Metastatic recurrence sites vary among the different breast cancer intrinsic subtypes [1, 3]. Distant metastases are frequently found during the post-operative course in patients with human epidermal growth factor receptor 2 (HER2)-enriched breast cancer and triple-negative breast cancer (TNBC), while they are relatively rare in patients with hormone receptor-positive and HER2-negative (HR+/HER2−) breast cancer. Therefore, early detection of locoregional recurrence after surgery for HR+/HER2− breast cancer has been suggested to be vital for good prognosis [7]. However, HR+/HER2− breast cancer can also be distant from the first site of recurrence.

Invasion and metastasis of cancer involve the molecular pathological features of not only the cancer but also the surrounding interstitial region, commonly called the tumor microenvironment (TME) [8, 9]. TME consists of cancer-related fibroblasts, neovascular cells, and tumor-infiltrating lymphocytes (TILs). Recently, a correlation between TILs and lymph node metastasis was reported in gastric cancer and melanoma [10, 11], and that between TILs and axillary lymph node metastasis was reported in TNBC [12, 13]. In all these reports, lower TIL density was shown to more likely cause lymph node metastasis. However, reports on the TIL relationship with distant metastases are few compared to those on its relationship with lymph node metastases. We speculate that if TILs are involved in lymph node metastasis, which is a risk factor for distant metastasis, TILs should also be involved in distant metastasis.

We hypothesized that the site of the first recurrence may vary based on TIL density in HR+/HER2− breast cancer. Therefore, this study aimed to investigate the correlation between TIL density and recurrence site in HR+/HER2− breast cancer, specifically in patients who did not receive chemotherapy either before or after surgery.

## Methods

### Patient background

In patients with stages I–II of HR+/HER2− breast cancer underwent surgery as the first treatment and received adjuvant endocrine therapy, except for adjuvant chemotherapy, between 2007 and 2015 at the Osaka City University Hospital, 42 patients were found to have recurrence and were enrolled in the present study. Breast cancers and their subtypes were diagnosed by core needle biopsy or vacuum-assisted biopsy. HR+/HER2− breast cancer was defined as estrogen receptor (ER)- and/or progesterone receptor (PgR)-positive and HER2-negative by immunohistological staining of the tissue. Expression of Ki67 was also examined using immunohistological staining and the cutoff value for Ki67 was set at 20% based on previous reports [14]. CT, US, and bone scintigraphy were used to evaluate the cancer stage. Cancer progression was evaluated according to the 8th edition of the TNM classification of malignant tumors (8th edition) by the Union for International Cancer Control [15]. All patients underwent either mastectomy or breast-conserving surgery, and the latter received radiation therapy in the remaining mammary gland post-surgery. For patients diagnosed with axillary lymph node metastasis by preoperative imaging, axillary lymph node dissection was also performed. In the case of breast cancer diagnosed without axillary lymph nodes metastasis, sentinel lymph node biopsy was performed using a combination of radioisotope and dye methods, as reported previously [16, 17]. Metastasis was examined pathologically by slicing sentinel lymph node at 2-mm thickness [18, 19]. Axillary lymph node dissection was performed in cases where metastasis in the sentinel lymph node was greater than 2 mm, also called macro-metastasis. The biological characteristics (ER/PgR/Ki67/HER2) of the resected specimens were re-examined, and the results validated those of the biopsy tissue. Adjuvant chemotherapy administration was decided at the discretion of the attending physician, taking into consideration the patient's wishes and comorbidities. In the present study, disease-free survival (DFS) was defined as the time from surgery to recurrence or death; progression-free survival (PFS) was defined as the time from recurrence to relapse or death due to breast cancer after the next treatment; post-recurrence survival (PRS) was defined as the time from recurrence to death due to breast cancer.

### Histopathological evaluation of TIL density

**Fig. 1:**
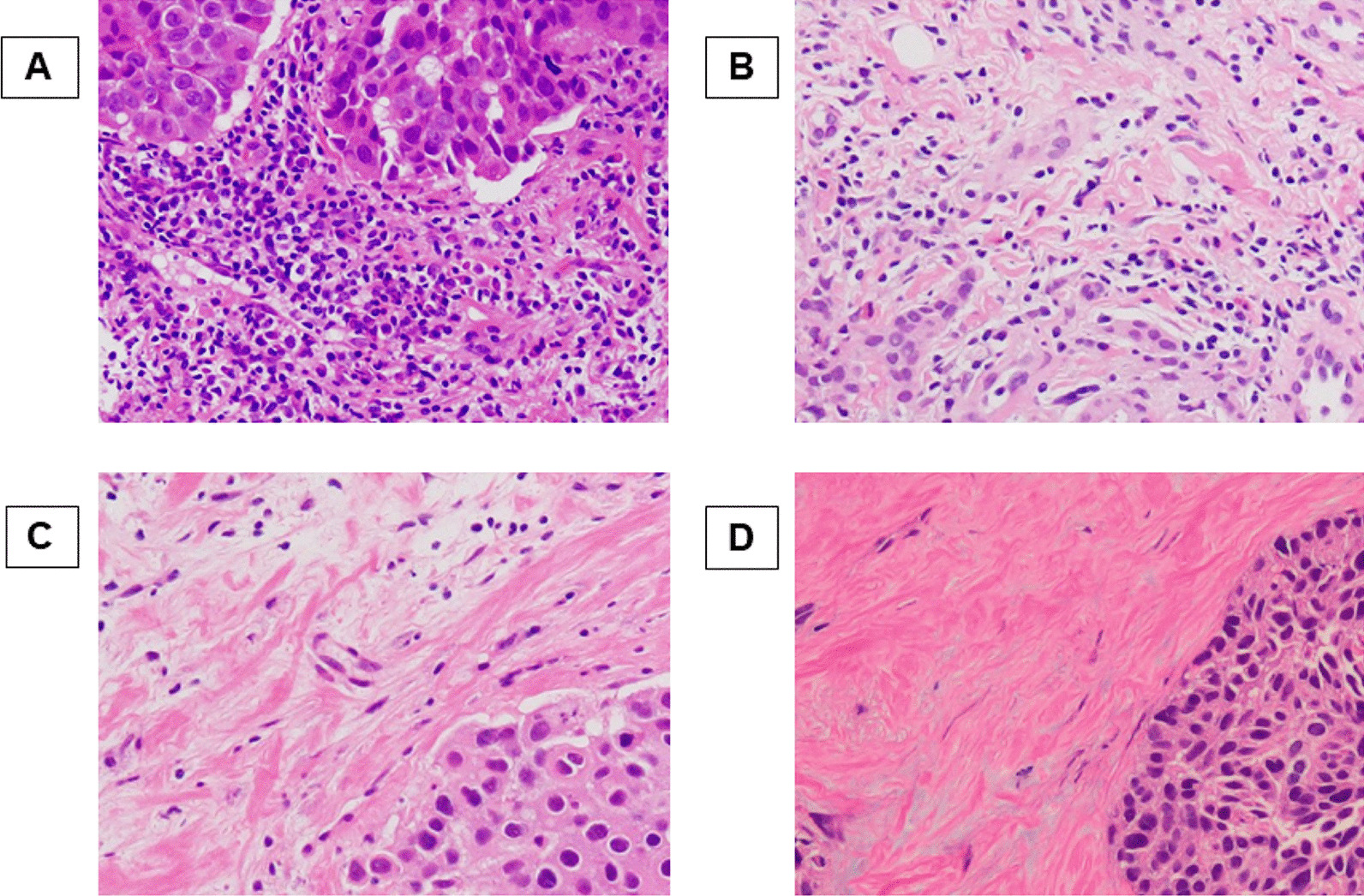
Histopathologic evaluation of the tumor-infiltrating lymphocytes (TILs) density was performed on hematoxylin and eosin-stained tumor section. The density of them was averaged on full sections, at least five fields, of the tumor stroma. The results were divided into four groups (> 50% (**A**), > 10–50% (**B**), ≤ 10% (**C**), and absent (**D**), respectively)

TIL density was evaluated pathologically using biopsy specimens. The pathological diagnosis and examination were jointly performed by two breast pathologists. The definition and evaluation method for TILs followed the International TILs Working Group 2014 [20]. Specifically, the density of infiltrating lymphocytes was averaged on full sections, at least five fields, of the tumor stroma. The results were divided into four groups according to previous reports (score 3: > 50%, score 2: > 10–50%, score 1: ≤ 10%, and score 0: absent) (Fig. 1) [21–24].

### Statistical analysis

All statistical analyses were performed using the JMP software package (SAS, Tokyo, Japan). For evaluating the correlation between two groups for each clinicopathological feature, Pearson’s chi-square test was used. The odds ratio (OR) and 95% confidence interval (CI) were calculated using logistic analysis. Multivariable analysis was performed with the multivariable logistic regression model. To evaluate the hazard ratio (HR) and 95% CI related to survival outcomes, such as PFS or PRS, Cox proportional hazards models were used for univariate analysis and Cox regression models were used for multivariate analysis. Significance was defined at *p* value < 0.05.

### Ethics statement

Written informed consent to participate in the study was obtained from each subject in accordance with the Declaration of Helsinki. Each patient or their family was fully informed of the nature of the investigation in this study prior to obtaining their written informed consent. The study protocol was approved by the Ethics Committee of Osaka City University (Approval No. # 926).

## Results

### Clinicopathological features

Table 1 shows the clinicopathological features of forty-two HR+/HER2− breast cancer patients with recurrence who had never received chemotherapy. The median age at the time of operation was 59 years (range 37–79 years). The median tumor size was 20.5 mm (9.5–49.3 mm), and no evidence of axillary lymph node metastasis was found by pre-operative imaging, in all cases. Therefore, sentinel lymph node biopsy was performed for all. Micro-metastasis was identified in three cases (7.1%) and macro-metastasis was found in seven cases (16.7%). The latter underwent axillary lymph node dissection, and pathological examination revealed < 3 axillary lymph nodes with metastases. The expression of ER in one patient (2.4%) was negative, while that of PgR was positive. Three patients (7.1%) were negative for PgR expression and seven (16.7%) had high Ki67. Eighteen cases (42.9%) underwent breast-conserving treatment and received post-operative radiation therapy for the residual mammary gland. None of the 42 patients received post-operative radiation therapy in the axilla or supraclavicular fossa. Post-operative pathological findings showed lymphatic invasion in 21 patients (50.0%) and venous invasion in three patients (7.1%). Six patients (14.3%) were diagnosed with nuclear grade 3. All patients received adjuvant endocrine therapy; 10 patients (23.8%) were treated with tamoxifen and seven patients (16.7%) were treated with a combination of tamoxifen and a luteinizing hormone-releasing hormone agonist. The remaining 25 patients (59.5%) received anastrozole. The median DFS time was 1462 days (range 132–3300 days). The first recurrence site was local in 18 (42.9%) patients and regional lymph node in 13 (30.9%) patients, while distant metastases were observed in 11 (26.2%) patients. Lung metastasis, detected in seven (16.6%) patients, was the most common distant metastasis. None of the patients showed simultaneous locoregional recurrence and distant metastasis. Six (14.3%) patients had TIL density higher than 10%, while in six other patients (14.3%), TIL density was not detected.

**Table 1:** Clinicopathological features of 42 patients with recurrence of hormone receptor-positive/HER2 negative breast cancer not receiving chemotherapy

Parameters	Number of all patients (n = 42) (%)
Age at operation (years old)	Median 59 (range 37–79)
Tumor size (mm)	Median 20.5 (range 9.5–49.3)
*Pathological lymph node metastasis*	
pN0/pN1mi/pN1a	32 (76.2%)/3 (7.1%)/7 (16.7%)
*Estrogen receptor*	
Negative/positive	1 (2.4%)/41 (97.6%)
*Progesterone receptor*	
Negative/positive	3 (7.1%)/39 (92.9%)
*Ki67*	
≤ 20%/ > 20%	35 (83.3%)/7 (16.7%)
*Surgical treatment*	
Breast conserving treatment and adjuvant radiation therapy/mastectomy	18 (42.9%)/24 (57.1%)
*Lymphatic invasion*	
ly0/ly1	21 (50.0%)/21 (50.0%)
*Venous invasion*	
v0/v1	39 (92.9%)/3 (7.1%)
*Nuclear grade*	
1/2/3	20 (47.6%)/16 (38.1%)/6 (14.3%)
*Adjuvant endocrine therapy*	
TAM/TAM + LH-RH agonist/ANA	10 (23.8%)/7 (16.7%)/25 (59.5%)
Disease free survival (days)	1462 (132–3300)
*Primary recurrence site*	
Local/regional lymph node/lung/bone/liver/bone + liver	18 (42.9%)/13 (30.9%)/7 (16.6%)/1 (2.4%)/1 (2.4%)/2 (4.8%)
*TILs density*	
Absent/ ≤ 10%/ > 10%	6 (14.3%)/30 (71.4%)/6 (14.3%)

*HER2* human epidermal growth factor receptor 2, *TAM* tamoxifen, *LH-RH* luteinizing hormone-releasing hormone, *ANA* anastrozole, *TILs* tumor-infiltrating lymphocytes

### Correlations between clinicopathological features and recurrence sites

The correlations between clinicopathological features and recurrence sites are listed in Table 2. According to sentinel lymph node biopsy results, two out of the seven cases with metastasis had regional lymph node recurrence and five had distant metastatic recurrence. Thus, in patients with axillary lymph node metastases, local recurrence was significantly less (*p* = 0.012), and distant metastasis was significantly more (*p* = 0.003) than that in patients without axillary lymph node metastases. In addition, in patients with longer DFS, distant metastatic recurrence was significantly higher (*p* = 0.014). Further, patients who underwent mastectomy had significantly more locoregional recurrence (*p* = 0.020). Examination of the correlations between operative procedures and clinicopathological features revealed the following significant differences in patients who underwent mastectomy: older age (*p* = 0.001), higher frequency of tumor with diameter more than 30 mm (*p* = 0.006), and lower Ki67 (*p* = 0.012) (Additional file 1: Table S1).

**Table 2:** Correlation between primary recurrence site and clinicopathological features in HR+/HER2− breast cancer not received chemotherapy

Parameters	Primary recurrence site (*n* = 42)					
	Local recurrence (*n* = 18)	Not local recurrence (*n* = 24)	*p* value	Locoregional recurrence (*n* = 31)	Distant metastasis (*n* = 11)	*p* value
*Age at operation (years old)*						
≤ 60	11 (61.1%)	14 (58.3%)	0.856	17 (54.8%)	8 (72.7%)	0.299
				14 (45.2%)	3 (27.3%)	
> 60	7 (38.9%)	10 (41.7%)				
*Tumor size (mm)*						
≤ 20.0	9 (50.0%)	12 (50.0%)	1.000	5 (48.4%)	6 (54.5%)	0.726
> 20.0	9 (50.0%)	12 (50.0%)		16 (51.6%)	5 (45.5%)	
*Tumor size (mm)*						
≤ 30.0	15 (83.3%)	19 (79.2%)	0.734	24 (77.4%)	10 (90.9%)	0.328
> 30.0	3 (16.7%)	5 (20.8%)		7 (22.6%)	1 (9.1%)	
*Pathological lymph node metastasis*						
pN0, pN1mi	18 (100.0%)	17 (70.8%)	0.012	29 (93.5%)	6 (54.5%)	0.003
pN1a	0 (0.0%)	7 (29.2%)		2 (6.5%)	5 (45.5%)	
*Progesterone receptor*						
Negative	2 (11.1%)	1 (4.2%)	0.387	3 (9.7%)	0 (0.0%)	0.284
Positive	16 (88.9%)	23 (95.8%)		28 (90.3%)	11 (100.0%)	
*Ki67*						
≤ 20%	16 (88.9%)	19 (79.2%)	0.403	26 (83.9%)	9 (81.8%)	0.875
> 20%	2 (11.1%)	5 (20.8%)		5 (16.1%)	2 (18.2%)	
*Surgical treatment*						
BCT and radiation therapy	7 (38.9%)	11 (45.8%)	0.653	0 (32.3%)	8 (72.7%)	0.020
Mastectomy	11 (61.1%)	13 (54.2%)		21 (67.7%)	3 (27.3%)	
*Lymphatic invasion*						
ly0	10 (55.6%)	11 (45.8%)	0.533	16 (51.6%)	5 (45.5%)	0.726
ly1	8 (44.4%)	13 (54.2%)		15 (48.4%)	6 (54.5%)	
*Venous invasion*						
v0	17 (94.4%)	22 (91.7%)	0.729	29 (93.5%)	10 (90.9%)	0.770
v1	1 (5.6%)	2 (8.3%)		2 (6.5%)	1 (9.1%)	
*Nuclear grade*						
1, 2	15 (83.3%)	21 (87.5%)	0.703	26 (83.9%)	10 (90.9%)	0.567
3	3 (16.7%)	3 (12.5%)		5 (16.1%)	1 (9.1%)	
*Adjuvant endocrine therapy*						
TAM (+ LH-RH agonist)	8 (44.4%)	9 (37.5%)	0.650	13 (41.9%)	4 (36.4%)	0.746
ANA	10 (55.6%)	15 (62.5%)		18 (58.1%)	7 (63.6%)	
*Disease free survival (days)*						
≤ 1462	12 (66.7%)	9 (37.5%)	0.061	19 (61.3%)	2 (18.2%)	0.014
> 1462	6 (33.3%)	15 (62.5%)		12 (38.7%)	9 (81.8%)	
*TILs density*						
≤ 10	15 (83.3%)	21 (87.5%)	0.703	27 (87.1%)	9 (81.8%)	0.667
> 10	3 (16.7%)	3 (12.5%)		4 (12.9%)	2 (18.2%)	
*TILs density*						
Absent	0 (0.0%)	6 (25.0%)	0.022	2 (6.5%)	4 (36.4%)	0.015
Not absent	18 (100.0%)	18 (75.0%)		29 (93.5%)	7 (63.6%)	

*HR*+*/HER2− breast cancer* hormone receptor-positive and human epidermal growth factor receptor 2 negative breast cancer, *BCT* breast conserving treatment, *TAM* tamoxifen, *LH-RH* luteinizing hormone-releasing hormone, *ANA* anastrozole, *TILs* tumor- infiltrating lymphocytes

Focusing on TIL density, no significant difference was observed when the cutoff value of TIL density was set at 10%. However, of the six patients with no TIL density, two had regional lymph node recurrence and four had distant metastatic recurrence. Therefore, in patients with no TIL density, local recurrence was significantly less (*p* = 0.022) and distant metastasis was significantly more (*p* = 0.015) than that in patients with TIL density.

### Correlations between clinicopathological features and TILs

We examined the correlation between clinicopathological features and TILs (Table 3); TIL density tended to be absent in patients with tumor size > 20 mm (*p* = 0.078) and 10% or more in patients with tumor size < 20 mm (*p* = 0.078). Macro-metastasis was found by sentinel lymph node biopsy in 4/6 patients with no TIL density. Significantly more lymph node metastasis was found in patients with no TIL than that in patients with TIL (*p* < 0.001). Significantly higher frequency of venous invasion was found in patients with TIL ≥ 10% than that in patients with TIL < 10% (*p* = 0.007).

**Table3:** Correlation between TILs and clinicopathological features in HR+/HER2− breast cancer not received chemotherapy

Parameters	Tumor- infiltrating lymphocytes (*n* = 42)					
	Absent (*n* = 6)	Not absent (*n* = 36)	*p* value	≤ 10 (*n* = 36)	> 10 (*n* = 6)	*p* value
*Age at operation (years old)*						
≤ 60 > 60	2 (33.3%) 4 (66.7%)	23 (63.9%) 13 (36.1%)	0.158	22 (61.1%) 14 (38.9%)	3 (50.0%) 3 (50.0%)	0.608
*Tumor size (mm)*						
≤ 20.0 > 20.0	1 (16.7%) 5 (83.3%)	20 (55.6%) 16 (44.4%)	0.078	16 (44.4%) 20 (55.6%)	5 (83.3%) 1 (16.7%)	0.078
*Tumor size (mm)*						
≤ 30.0 > 30.0	4 (66.7%) 2 (33.3%)	30 (83.3%) 6 (16.7%)	0.336	28 (77.8%) 8 (22.2%)	6 (100.0%) 0 (0.0%)	0.199
*Pathological lymph node metastasis*						
pN0, pN1mi pN1a	2 (33.3%) 4 (66.7%)	33 (91.7%) 3 (8.3%)	< 0.001	29 (80.6%) 7 (19.4%)	6 (100.0%) 0 (0.0%)	0.237
*Progesterone receptor*						
Negative Positive	1 (16.7%) 5 (83.3%)	2 (5.6%) 34 (94.4%)	0.328	2 (5.6%) 34 (94.4%)	1 (16.7%) 5 (83.3%)	0.328
*Ki67*						
≤ 20% > 20%	6 (100.0%) 0 (0.0%)	29 (80.6%) 7 (19.4%)	0.237	29 (80.6%) 7 (19.4%)	6 (100.0%) 0 (0.0%)	0.237
*Surgical treatment*						
BCT and radiation therapy Mastectomy	2 (33.3%) 4 (66.7%)	16 (44.4%) 20 (55.6%)	0.611	15 (41.7%) 21 (58.3%)	3 (50.0%) 3 (50.0%)	0.703
Lymphatic invasion						
ly0 ly1	3 (50.0%) 3 (50.0%)	18 (50.0%) 18 (50.0%)	1.000	18 (50.0%) 18 (50.0%)	3 (50.0%) 3 (50.0%)	1.000
*Venous invasion*						
v0 v1	6 (100.0%) 0 (0.0%)	33 (91.7%) 3 (8.3%)	0.463	35 (97.2%) 1 (2.8%)	4 (66.7%) 2 (33.3%)	0.007
*Nuclear grade*						
1, 2 3	5 (83.3%) 1 (16.7%)	1 (86.1%) 5 (13.9%)	0.857	31 (86.1%) 5 (13.9%)	5 (83.3%) 1 (16.7%)	0.857
*Adjuvant endocrine therapy*						
TAM (+ LH-RH agonist) ANA	1 (16.7%) 5 (83.3%)	16 (44.4%) 20 (55.6%)	0.199	15 (41.7%) 21 (58.3%)	2 (33.3%) 4 (66.7%)	0.700
*Disease free survival (days)*						
≤ 1462 > 1462	1 (16.7%) 5 (83.3%)	20 (55.6%) 16 (44.4%)	0.078	18 (50.0%) 18 (50.0%)	3 (50.0%) 3 (50.0%)	1.000
*Primary recurrence site*						
Local recurrence Not local recurrence	0 (0.0%) 6 (100.0%)	8 (50.0%) 18 (50.0%)	0.022	5 (41.7%) 21 (58.3%)	3 (50.0%) 3 (50.0%)	0.703
*Primary recurrence site*						
Locoregional recurrence Distant metastasis	2 (33.3%) 4 (66.7%)	29 (80.6%) 7 (19.4%)	0.015	27 (75.0%) 9 (25.0%)	4 (66.7%) 2 (33.3%)	0.667

*TILs* tumor- infiltrating lymphocytes, *HR*+*/HER2− breast cancer* hormone receptor-positive and human epidermal growth factor receptor 2 negative breast cancer, *BCT* breast conserving treatment, *TAM* tamoxifen, *LH-RH* luteinizing hormone-releasing hormone, *ANA* anastrozole, *TILs* tumor-infiltrating lymphocytes

### Prediction of distant metastases in patients with HR+/HER2− breast cancer without chemotherapy

Examining distant metastasis predictors based on the results, axillary lymph node metastasis (*p* = 0.003, OR = 16.723) and operative method (*p* = 0.026, OR = 0.044) were found to be independent factors (Table 4). TILs (absent vs. present) were predictors of distant metastases as per univariate analysis (*p* = 0.015, OR = 0.127), though not independent factors (*p* = 0.285, OR = 0.144).

**Table 4:** Univariate and multivariate analysis with distant metastasis for HR+/HER2− breast cancer not received chemotherapy

Parameters	Univarite analysis			Multivarite analysis		
	Odd ratio	95% CI	*p* value	Odd ratio	95% CI	*p* value
*Age at operation (years old)*						
≤ 60 versus > 60	0.455	0.101–2.048	0.299			
*Tumor size (mm)*						
≤ 20.0 versus > 20.0	0.781	0.197–3.106	0.726			
*Tumor size (mm)*						
≤ 30.0 versus > 30.0	0.343	0.037–3.161	0.328			
*Pathological lymph node metastasis*						
pN0, pN1mi versus pN1a	12.083	1.880–77.665	0.003	16.723	1.197–520.348	0.050
*Progesterone receptor*						
Negative versus positive	–	–	0.284			
*Ki67*						
≤ 20% versus > 20%	1.156	0.190–7.037	0.875			
*Surgical treatment*						
BCT and radiation therapy versus mastectomy	0.179	0.039–0.821	0.020	0.044	0.001–0.420	0.026
*Lymphatic invasion*						
ly0 versus ly1	1.280	0.322–5.088	0.726			
*Venous invasion*						
v0 versus v1	1.450	0.118–17.766	0.770			
*Nuclear grade*						
1, 2 versus 3	0.520	0.054–5.021	0.567			
*Adjuvant endocrine therapy*						
TAM (+ LH-RH agonist) versus ANA	0.867	0.217–3.461	0.839			
*Disease free survival (days)*						
≤ 1462 versus > 1462	7.125	1.309–38.771	0.014	6.693	0.954–81.406	0.080
*TILs density*						
≤ 10 versus > 10	1.500	0.234–9.611	0.667			
*TILs*						
Absent versus Not absent	0.127	0.018–0.797	0.015	0.144	0.003–5.060	0.285

*HR*+*/HER2− breast cancer* hormone receptor-positive and human epidermal growth factor receptor 2 negative breast cancer, *CI* confidence intervals, *BCT* breast conserving treatment, *TAM* tamoxifen, *LH-RH* luteinizing hormone-releasing hormone, *ANA* anastrozole, *TILs* tumor- infiltrating lymphocytes

### Prognosis after recurrence

Using univariate analysis with PFS after recurrence, no clear predictive factor could be identified, since treatment after recurrence was variable (Additional file 2: Table S2). On the other hand, as per multivariate analysis with PRS, lymph node metastasis during surgery was found to be a poor prognostic factor (*p* = 0.042, HR = 17.339) (Table 5).

**Table 5:** Univariate and multivariate analysis with post-recurrence survival after recurrence

Parameters	Univarite analysis			Multivarite analysis		
	Hazard ratio	95% CI	*p* value	Hazard ratio	95% CI	*p* value
*Age at operation (years old)*						
≤ 60 versus > 60	0.871	0.040–9.101	0.910			
*Tumor size (mm)*						
≤ 20.0 versus > 20.0	1.798	0.172–38.728	0.623			
*Tumor size (mm)*						
≤ 30.0 versus > 30.0	–	–	0.265			
*Pathological lymph node metastasis*						
pN0, pN1mi versus pN1a	21.520	1.878–488.577	0.016	17.339	1.112–530.855	0.042
*Progesterone receptor*						
Negative versus positive	–	–	0.545			
*Ki67*						
≤ 20% versus > 20%	1.806	0.084–18.864	0.642			
*Surgical treatment*						
BCT and radiation therapy versus mastectomy	0.422	0.020–4.410	0.467			
*Lymphatic invasion*						
ly0 versus ly1	1.906	0.182–41.094	0.589			
*Venous invasion*						
v0 versus v1	3.760	0.174–39.725	0.327			
*Nuclear grade*						
1, 2 versus 3	–	–	0.294			
*Adjuvant endocrine therapy*						
TAM (+ LH-RH agonist) versus ANA	0.422	0.020–4.410	0.467			
*Disease free survival (days)*						
≤ 1462 versus > 1462	1.229	0.056–13.152	0.870			
*Primary recurrence site*						
Locoregional recurrence versus Distant metastasis	9.331	0.877–202.892	0.063	6.057	0.414–178.741	0.187
*TILs density*						
≤ 10 versus > 10	3.405	0.158–35.582	0.360			
*TILs*						
Absent versus Not absent	0.211	0.018–4.800	0.269			

*CI* confidence intervals, *BCT* breast conserving treatment, *TAM* tamoxifen, *LH-RH* luteinizing hormone-releasing hormone, *ANA* anastrozole, *TILs* tumor- infiltrating lymphocytes

## Discussion

There are various reports on the prediction of distant metastasis; however, the recurrence site in these reports differs depending on the subtype, and tumor size and lymph node metastasis are common risk factors [2, 25–27]. In addition, young age [2], histopathological grade, and lymphovascular invasion have been reported as risk factors [2, 26], although there are reports suggesting otherwise, as well. In this study, a combination of “BCT and radiation therapy” as post-operative procedures was found to be predictive factor for distant metastasis. The predictive factor status may be attributed to the fact that “BCT and radiation therapy” group was significantly younger, and had larger tumor diameter and higher Ki67 compared to the “mastectomy” group. They are the risk factors listed above. Although each parameter alone was not a predictor, they were found to turn into a predictor when combined.

Formation of distant metastases in cancer generally follows the concept of “seed and soil.”[28]. TME corresponds to the “soil” as per this concept [8, 9]. TILs are also included in the cells that constitute the TME. In breast cancer, TILs have been reported to vary by subtype. In particular, HER2-enriched breast cancer and TNBC have been reported to show significantly higher TIL density than HR+/HER2− breast cancer, and TILs have been proven to predict the therapeutic effect of chemotherapy [13, 29–31]. In contrast, there are very few reports examining the correlation between TILs and clinicopathological factors or therapeutic effects in HR+/HER2− breast cancer.

In this study, we excluded patients who had undergone neoadjuvant/adjuvant chemotherapy based on three reasons. First, chemotherapy affects the immune microenvironment, including TILs, in preoperative chemotherapy study, and related changes may affect prognosis [32]. TILs are also predictors of the therapeutic effect of adjuvant chemotherapy; therefore, adjuvant chemotherapy may also affect the tumor immune microenvironment. Secondly, axillary lymph node metastasis before adjuvant chemotherapy was diagnosed based on image only, and therefore, the diagnosis was not accurate. Lastly, different chemotherapy regimens are known to have different effects, and neoadjuvant chemotherapy causes more local recurrence than adjuvant therapy [33].

There are some reports on the relationship between recurrence sites and TILs. Park et al. [34] reported patients with TILs > 10% in early-stage TNBC to show significantly more locoregional recurrence than those with lower TILs. In cervical squamous cell carcinoma, low TILs are also likely to cause distant metastasis [35]. Moreover, distant metastasis is reportedly predicted from pathological features, including lymphocytes of lymph nodes in breast cancer with lymph node metastasis, although not from the density of lymphocytes around the tumor [36]. Bidwell et al. [37] have shown innate immune escape to promote bone metastasis, based on clinical data and experiments in mice. Some studies using breast cancer cell lines in vivo also reported that immunosuppression in the tumor immune environment increased the risk of lung metastasis [38, 39]. In this study, TIL density was suggested to possibly be a distant metastatic predictor, although not an independent factor. We previously reported that TILs may also be involved in lymph node metastasis in HR+/HER2− breast cancer [40]. In this study, TILs were considered to be strongly correlated with lymph node metastasis, and were involved in distant metastasis prediction as well as prognosis after recurrence.

The greatest limitation of this study was that very few cases of recurrence were examined. Another limitation was that the patients received different types of adjuvant endocrine therapy. It will be necessary to examine by accumulating future cases. Moreover, younger and lymphovascular invasion, which were previously reported as risk factors, were not found to be predictors in this study. TILs may also be involved in venous invasion. Furthermore, considering that the TIL subtypes have different functions, detailed studies are necessary to identify the organs that are prone to metastasis. Immunohistochemical staining is needed to determine TILs subtypes. Nevertheless, TILs can be easily evaluated using needle biopsy specimens for the diagnosis of breast cancer and are highly useful. Filipits et al. [41] predicted distant metastases using a combination of the above-mentioned predictors and TILs may be considered to be an additional predictor. Currently, postoperative follow-up involves patient interview, palpation, and mammography. Whole-body imaging for asymptomatic breast cancer patients post resection surgery is not recommended [4–6]. However, late diagnosis of distant metastasis, that is, after emergence of detectable symptoms, leads to impairment of the patient’s quality of life. Moreover, the risk of reduced treatment options, owing to the deterioration in the general condition as a result of recurrence, prevails. Therefore, it is important to identify and diagnose breast cancer that is prone to distant metastasis.

## Conclusions

In stages I–II of HR+/HER2− breast cancer, locoregional recurrence is common and distant metastasis is relatively rare. This study suggested TILs to possibly be one of predictors of distant metastatic recurrence in stages I–II of HR+/HER2− breast cancer in patients who do not undergo chemotherapy.

## Supplementary Information

**Additional file 1: Table S1**. Correlation between surgical treatment and clinicopathological features in HR+/HER2 breast cancer not received chemotherapy.

**Additional file 2: Table S2**. Univariate analysis with progression free survival after recurrence.

## Data Availability

The datasets used and/or analyzed during the current study are available from the corresponding author on reasonable request.

## Abbreviations

BC: Breast cancer
CI: Confidence interval
CT: Computed tomography
CTCs: Circulating tumor cells
DFS: Disease free survival
DNA: Deoxyribonucleic acid
ER: Estrogen receptor
HER2: Human epidermal growth factor receptor 2
HR+/HER2− breast cancer: Hormone receptor-positive and HER2-negative breast cancer
HRs: Hazard ratios
Net1: Neuroepithelioma transforming gene 1
OR: Odd ratio
PFS: Progression-free survival
PgR: Progesterone receptor
PRS: Post-recurrence survival
TNBC: Triple-negative breast cancer
TILs: Tumor infiltrating lymphocytes
TME: Tumor microenvironment
US: Ultrasonography

## References

[1] Chen W, Hoffmann AD, Liu H, Liu X (2018). Organotropism: new insights into molecular mechanisms of breast cancer metastasis. NPJ Precis Oncol.

[2] Lim YJ, Lee SW, Choi N, Kwon J, Eom KY, Kang E, Kim EK, Kim JH, Kim YJ, Kim SH (2018). A novel prognostic nomogram for predicting risks of distant failure in patients with invasive breast cancer following postoperative adjuvant radiotherapy. Cancer Res Treat.

[3] Wu Q, Li J, Zhu S, Wu J, Chen C, Liu Q, Wei W, Zhang Y, Sun S (2017). Breast cancer subtypes predict the preferential site of distant metastases: a SEER based study. Oncotarget.

[4] Ghezzi P, Magnanini S, Rinaldini M, Berardi F, Di Biagio G, Testare F, Tavoni N, Schittulli F, D'Amico C, Pedicini T, Fumagalli M (1994). Impact of follow-up testing on survival and health-related quality of life in breast cancer patients. A multicenter randomized controlled trial. The GIVIO Investigators. JAMA.

[5] Rosselli Del Turco M, Palli D, Cariddi A, Ciatto S, Pacini P, Distante V (1994). Intensive diagnostic follow-up after treatment of primary breast cancer. A randomized trial. National Research Council Project on Breast Cancer follow-up. JAMA.

[6] Palli D, Russo A, Saieva C, Ciatto S, Rosselli Del Turco M, Distante V, Pacini P (1999). Intensive vs clinical follow-up after treatment of primary breast cancer: 10-year update of a randomized trial. National Research Council Project on Breast Cancer Follow-up. JAMA.

[7] Puglisi F, Fontanella C, Numico G, Sini V, Evangelista L, Monetti F, Gori S, Del Mastro L (2014). Follow-up of patients with early breast cancer: is it time to rewrite the story?. Crit Rev Oncol Hematol.

[8] Soysal SD, Tzankov A, Muenst SE (2015). Role of the tumor microenvironment in breast cancer. Pathobiology.

[9] Hanahan D, Coussens LM (2012). Accessories to the crime: functions of cells recruited to the tumor microenvironment. Cancer Cell.

[10] Kim JY, Kim CH, Lee Y, Lee JH, Chae YS (2017). Tumour infiltrating lymphocytes are predictors of lymph node metastasis in early gastric cancers. Pathology.

[11] Azimi F, Scolyer RA, Rumcheva P, Moncrieff M, Murali R, McCarthy SW, Saw RP, Thompson JF (2012). Tumor-infiltrating lymphocyte grade is an independent predictor of sentinel lymph node status and survival in patients with cutaneous melanoma. J Clin Oncol.

[12] Caziuc A, Schlanger D, Amarinei G, Dindelegan GC (2019). Can tumor-infiltrating lymphocytes (TILs) be a predictive factor for lymph nodes status in both early stage and locally advanced breast Cancer?. J Clin Med.

[13] Adams S, Gray RJ, Demaria S, Goldstein L, Perez EA, Shulman LN, Martino S, Wang M, Jones VE, Saphner TJ (2014). Prognostic value of tumor-infiltrating lymphocytes in triple-negative breast cancers from two phase III randomized adjuvant breast cancer trials: ECOG 2197 and ECOG 1199. J Clin Oncol.

[14] Goldhirsch A, Winer EP, Coates AS, Gelber RD, Piccart-Gebhart M, Thürlimann B, Senn H-J (2013). Panel members: Personalizing the treatment of women with early breast cancer: highlights of the St Gallen International Expert Consensus on the Primary Therapy of Early Breast Cancer 2013. Ann Oncol.

[15] Cserni G, Chmielik E, Cserni B, Tot T (2018). The new TNM-based staging of breast cancer. Virchows Arch.

[16] McMasters KM, Tuttle TM, Carlson DJ, Brown CM, Noyes RD, Glaser RL, Vennekotter DJ, Turk PS, Tate PS, Sardi A (2000). Sentinel lymph node biopsy for breast cancer: a suitable alternative to routine axillary dissection in multi-institutional practice when optimal technique is used. J Clin Oncol.

[17] Kashiwagi S, Onoda N, Asano Y, Kurata K, Noda S, Kawajiri H, Takashima T, Ohsawa M, Kitagawa S, Hirakawa K (2015). Ambulatory sentinel lymph node biopsy preceding neoadjuvant therapy in patients with operable breast cancer: a preliminary study. World J Surg Oncol.

[18] Lee A, Krishnamurthy S, Sahin A, Symmans WF, Hunt K, Sneige N (2002). Intraoperative touch imprint of sentinel lymph nodes in breast carcinoma patients. Cancer.

[19] Khanna R, Bhadani S, Khanna S, Pandey M, Kumar M (2011). Touch imprint cytology evaluation of sentinel lymph node in breast cancer. World J Surg.

[20] Salgado R, Denkert C, Demaria S, Sirtaine N, Klauschen F, Pruneri G, Wienert S, Van den Eynden G, Baehner FL, Penault-Llorca F (2015). The evaluation of tumor-infiltrating lymphocytes (TILs) in breast cancer: recommendations by an International TILs Working Group 2014. Ann Oncol.

[21] Kashiwagi S, Asano Y, Goto W, Takada K, Takahashi K, Noda S, Takashima T, Onoda N, Tomita S, Ohsawa M (2017). Use of Tumor-infiltrating lymphocytes (TILs) to predict the treatment response to eribulin chemotherapy in breast cancer. PLoS ONE.

[22] Ono M, Tsuda H, Shimizu C, Yamamoto S, Shibata T, Yamamoto H, Hirata T, Yonemori K, Ando M, Tamura K (2012). Tumor-infiltrating lymphocytes are correlated with response to neoadjuvant chemotherapy in triple-negative breast cancer. Breast Cancer Res Treat.

[23] Asano Y, Kashiwagi S, Goto W, Takada K, Takahashi K, Hatano T, Takashima T, Tomita S, Motomura H, Ohsawa M (2018). Prediction of treatment response to neoadjuvant chemotherapy in breast cancer by subtype using tumor-infiltrating lymphocytes. Anticancer Res.

[24] Kashiwagi S, Asano Y, Goto W, Takada K, Morisaki T, Kouhashi R, Yabumoto A, Tanaka S, Takashima T, Ohsawa M (2020). Validation of systemic and local tumour immune response to eribulin chemotherapy in the treatment of breast cancer. Anticancer Res.

[25] Arriagada R, Rutqvist LE, Johansson H, Kramar A, Rotstein S (2008). Predicting distant dissemination in patients with early breast cancer. Acta Oncol.

[26] Holleczek B, Stegmaier C, Radosa JC, Solomayer EF, Brenner H (2019). Risk of loco-regional recurrence and distant metastases of patients with invasive breast cancer up to ten years after diagnosis - results from a registry-based study from Germany. BMC Cancer.

[27] Koscielny S, Tubiana M, Le MG, Valleron AJ, Mouriesse H, Contesso G, Sarrazin D (1984). Breast cancer: relationship between the size of the primary tumour and the probability of metastatic dissemination. Br J Cancer.

[28] Paget S (1989). The distribution of secondary growths in cancer of the breast. Cancer Metastasis Rev.

[29] Denkert C, von Minckwitz G, Brase JC, Sinn BV, Gade S, Kronenwett R, Pfitzner BM, Salat C, Loi S, Schmitt WD (2015). Tumor-infiltrating lymphocytes and response to neoadjuvant chemotherapy with or without carboplatin in human epidermal growth factor receptor 2-positive and triple-negative primary breast cancers. J Clin Oncol.

[30] Ohtani H, Mori-Shiraishi K, Nakajima M, Ueki H (2015). Defining lymphocyte-predominant breast cancer by the proportion of lymphocyte-rich stroma and its significance in routine histopathological diagnosis. Pathol Int.

[31] Stanton SE, Adams S, Disis ML (2016). Variation in the incidence and magnitude of tumor-infiltrating lymphocytes in breast cancer subtypes: a systematic review. JAMA Oncol.

[32] Goto W, Kashiwagi S, Asano Y, Takada K, Takahashi K, Hatano T, Takashima T, Tomita S, Motomura H, Ohsawa M (2018). Predictive value of improvement in the immune tumour microenvironment in patients with breast cancer treated with neoadjuvant chemotherapy. ESMO Open.

[33] Early Breast Cancer Trialists’ Collaborative G (2018). Long-term outcomes for neoadjuvant versus adjuvant chemotherapy in early breast cancer: meta-analysis of individual patient data from ten randomised trials. Lancet Oncol.

[34] Park JH, Lee HJ, Lee SB, Ahn JH, Kim JE, Jung KH, Gong G, Son BH, Ahn SH, Kim SB (2019). Intrinsic prognostic impact of tumor-infiltrating lymphocytes in systemically untreated patients with early-stage triple-negative breast cancer. Anticancer Res.

[35] Cao L, Sun PL, He Y, Yao M, Gao H (2020). Immune stromal features in cervical squamous cell carcinoma are prognostic factors for distant metastasis: a retrospective study. Pathol Res Pract.

[36] Grigoriadis A, Gazinska P, Pai T, Irhsad S, Wu Y, Millis R, Naidoo K, Owen J, Gillett CE, Tutt A (2018). Histological scoring of immune and stromal features in breast and axillary lymph nodes is prognostic for distant metastasis in lymph node-positive breast cancers. J Pathol Clin Res.

[37] Bidwell BN, Slaney CY, Withana NP, Forster S, Cao Y, Loi S, Andrews D, Mikeska T, Mangan NE, Samarajiwa SA (2012). Silencing of Irf7 pathways in breast cancer cells promotes bone metastasis through immune escape. Nat Med.

[38] Na YR, Yoon YN, Son DI, Seok SH (2013). Cyclooxygenase-2 inhibition blocks M2 macrophage differentiation and suppresses metastasis in murine breast cancer model. PLoS ONE.

[39] Olkhanud PB, Damdinsuren B, Bodogai M, Gress RE, Sen R, Wejksza K, Malchinkhuu E, Wersto RP, Biragyn A (2011). Tumor-evoked regulatory B cells promote breast cancer metastasis by converting resting CD4(+) T cells to T-regulatory cells. Cancer Res.

[40] Takada K, Kashiwagi S, Asano Y, Goto W, Kouhashi R, Yabumoto A, Morisaki T, Shibutani M, Takashima T, Fujita H (2020). Prediction of lymph node metastasis by tumor-infiltrating lymphocytes in T1 breast cancer. BMC Cancer.

[41] Filipits M, Rudas M, Jakesz R, Dubsky P, Fitzal F, Singer CF, Dietze O, Greil R, Jelen A, Sevelda P (2011). A new molecular predictor of distant recurrence in ER-positive, HER2-negative breast cancer adds independent information to conventional clinical risk factors. Clin Cancer Res.

