# Supplementary material for: Prediction of distant metastatic recurrence by tumor-infiltrating lymphocytes in hormone receptor-positive breast cancer
Source: BMC Womens Health. 2021 May 29;21:225. doi: 10.1186/s12905-021-01373-7 (PMC8164786; doi:10.1186/s12905-021-01373-7)
Supplement: Supplementary file 1 — Additional file 1: Table S1. Correlation between surgical treatment and clinicopathological features in HR+/HER2 breast cancer not received chemotherapy. [file 12905_2021_1373_MOESM1_ESM.docx]

**Additional file 1; Table S1. Correlation between surgical treatment and clinicopathological features in HR+/HER2- breast cancer not received chemotherapy.**

| Parameters | Surgical treatment (*n* = 42) | | |
| --- | --- | --- | --- |
|  | BCT and radiation therapy  (*n* =18) | Mastectomy  (*n* =24) | *p* value |
| Age at operation (years old)  ≤ 60  > 60 | 16 (88.9 %)  2 (11.1 %) | 9 (37.5 %)  15 (62.5 %) | 0.001 |
| Tumor size (mm)  ≤ 20.0  > 20.0 | 11 (61.1 %)  7 (38.9 %) | 10 (41.7 %)  14 (58.3 %) | 0.212 |
| Tumor size (mm)  ≤ 30.0  > 30.0 | 18 (100.0 %)  0 (0.0 %) | 16 (66.7 %)  8 (33.3 %) | 0.006 |
| Pathological lymph node metastasis  pN0, pN1mi  pN1a | 15 (83.3 %)  3 (16.7 %) | 20 (83.3 %)  4 (16.7 %) | 1.000 |
| Progesterone receptor  Negative  Positive | 0 (0.0 %)  18 (100.0 %) | 3 (12.5 %)  21 (87.5 %) | 0.120 |
| Ki67  ≤20 %  >20 % | 12 (66.7 %)  6 (33.3 %) | 23 (95.8 %)  1 (4.2 %) | 0.012 |
| Lymphatic invasion  ly0  ly1 | 7 (38.9 %)  11 (61.1 %) | 14 (58.3 %)  10 (41.7 %) | 0.212 |
| Venous invasion  v0  v1 | 17 (94.4 %)  1 (5.6 %) | 22 (91.7 %)  2 (8.3 %) | 0.729 |
| Nuclear grade  1, 2  3 | 17 (94.4 %)  1 (5.6 %) | 19 (79.2 %)  5 (20.8 %) | 0.161 |
| Adjuvant endocrine therapy  TAM (+ LH-RH agonist)  ANA | 10 (55.6 %)  8 (44.4 %) | 7 (29.2 %)  17 (70.8 %) | 0.085 |
| Disease free survival (days)  ≤1462  >1462 | 11 (61.1 %)  7 (38.9 %) | 10 (41.7 %)  14 (58.3 %) | 0.212 |
| Primary recurrence site  Local recurrence  Not local recurrence | 7 (38.9 %)  11 (61.1 %) | 11 (45.8 %)  13 (54.2 %) | 0.653 |
| Primary recurrence site  Locoregional recurrence  Distant metastasis | 10 (55.6 %)  8 (44.4 %) | 21 (87.5 %)  3 (12.5 %) | 0.020 |
| TILs density  ≤10  >10 | 15 (83.3 %)  3 (16.7 %) | 21 (87.5 %)  3 (12.5 %) | 0.703 |
| TILs density  Absent  Not absent | 2 (11.1 %)  16 (88.9 %) | 4 (16.7 %)  20 (83.3 %) | 0.611 |

HR+/HER2- breast cancer: hormone receptor-positive and human epidermal growth factor receptor 2 negative breast cancer. BCT: breast conserving treatment. TAM: tamoxifen. LH-RH: luteinizing hormone-releasing hormone. ANA: anastrozole. TILs: tumor- infiltrating lymphocytes.
