# Supplementary material for: Prediction of distant metastatic recurrence by tumor-infiltrating lymphocytes in hormone receptor-positive breast cancer
Source: BMC Womens Health. 2021 May 29;21:225. doi: 10.1186/s12905-021-01373-7 (PMC8164786; doi:10.1186/s12905-021-01373-7)
Supplement: Supplementary file 2 — Additional file 2: Table S2. Univariate analysis with progression free survival after recurrence. [file 12905_2021_1373_MOESM2_ESM.docx]

**Additional file 2; Table S2. Univariate analysis with progression-free survival after recurrence.**

|  | Univarite analysis | | |
| --- | --- | --- | --- |
| Parameters | Hazard ratio | 95 % CI | *p* value |
| Age at operation (years old)  ≤ 60 vs > 60 | 0.599 | 0.190-1.620 | 0.322 |
| Tumor size (mm)  ≤ 20.0 vs > 20.0 | 1.696 | 0.647-4.707 | 0.283 |
| Tumor size (mm)  ≤ 30.0 vs > 30.0 | 2.034 | 0.562-5.968 | 0.253 |
| Pathological lymph node metastasis  pN0, pN1mi vs pN1a | 1.440 | 0.328-4.539 | 0.586 |
| Progesterone receptor  Negative vs Positive | 0.482 | 0.131-3.103 | 0.382 |
| Ki67  ≤20 % vs >20 % | 0.844 | 0.194-2.612 | 0.788 |
| Surgical treatment  BCT and radiation therapy vs Mastectomy | 0.962 | 0.367-2.565 | 0.936 |
| Lymphatic invasion  ly0 vs ly1 | 1.119 | 0.414-2.983 | 0.820 |
| Venous invasion  v0 vs v1 | 2.546 | 0.398-9.248 | 0.273 |
| Nuclear grade  1, 2 vs 3 | 0.312 | 0.017-1.530 | 0.178 |
| Adjuvant endocrine therapy  TAM (+ LH-RH agonist) vs ANA | 0.842 | 0.321-2.245 | 0.724 |
| Disease free survival (days)  ≤1462 vs >1462 | 1.174 | 0.437-3.100 | 0.744 |
| Primary recurrence site  Locoregional recurrence vs Distant metastasis | 1.778 | 0.552-5.030 | 0.313 |
| TILs density  ≤10 vs >10 | 2.151 | 0.475-0.727 | 0.286 |
| TILs  Absent vs Not absent | 1.362 | 0.375-8.717 | 0.674 |

CI, confidence intervals. BCT: breast conserving treatment. TAM: tamoxifen. LH-RH: luteinizing hormone-releasing hormone. ANA: anastrozole. TILs: tumor- infiltrating lymphocytes.
